# Understanding caregiver burden and quality of life in Kerala’s primary palliative care program: a mixed methods study from caregivers and providers’ perspectives

**DOI:** 10.1186/s12939-024-02155-x

**Published:** 2024-05-07

**Authors:** Arsha Kochuvilayil, Ravi Prasad Varma

**Affiliations:** Achutha Menon Centre for Health Science Studies, Sree Chitra Tirunal Institute for Medical Sciences and Technology, Trivandrum, Thiruvananthapuram, Kerala India

**Keywords:** Family caregivers, Ageing, Caregiver burden, Quality of life, Utility scores, Palliative nurse, Palliative care

## Abstract

**Background:**

Family caregivers are vital for long-term care for persons with serious health-related suffering in Kerala. Long-term caregiving and ageing may become burdensome and detrimental to patients and caregivers. We compared the caregiver burden and quality-of-life of ageing caregivers with younger caregivers. We also explored the palliative care nurses’ perceptions of the family caregivers’ issues.

**Methods:**

We did a mixed method study focusing on two groups: (i) three in-depth interviews and a cross-sectional survey among 221 caregivers of palliative care patients in five randomly selected panchayats (most peripheral tier of three-tier local self-government system in India concerned with governance of a village or small town) of Kollam district, Kerala, as part of development and validation of the Achutha Menon Centre Caregiver Burden Inventory; (ii) five in-depth interviews with purposively selected primary palliative care nurses as part of a study on local governments and palliative care. We used a structured interview schedule to collect cross-sectional data on sociodemographic and caregiving-related characteristics, caregiver burden, and health-related quality of life using the EuroQol EQ5D5L and interview guidelines on caregiver issues tailored based on participant type for qualitative interviews.

**Results:**

Older caregivers comprised 28.1% of the sample and had significantly poorer health and quality-of-life attributes. More senior caregivers experiencing caregiver burden had the lowest mean scores of 0.877 (Standard deviation (SD 0.066, 95% confidence intervals (CI) 0.854–0.899) followed by younger caregivers with high burden (0.926, SD 0.090, 95% CI 0.907–0.945), older caregivers with low burden (0.935, SD 0.058, 95% CI 0.912–0.958) and younger caregivers with low burden (0.980, SD 0.041, 95% CI 0.970–0.990). Caregivers faced physical, psychological, social, and financial issues, leading to a caregiver burden. The relationships between the palliative care nurses and family caregivers were complex, and nurses perceived caregiver burden, but there were no specific interventions to address this.

**Conclusion:**

In our study from Kollam, Kerala, three out of ten caregivers of palliative care patients were 60 years of age or older. They had significantly lower health-related quality of life, particularly if they perceived caregiver burden. Despite being recognized by palliative care nurses, caregiver issues were not systematically addressed. Further research and suitable interventions must be developed to target such problems in the palliative care programme in Kerala.

**Supplementary Information:**

The online version contains supplementary material available at 10.1186/s12939-024-02155-x.

## Background

Norman Daniels “We should not allow misfortune to beget injustice” [1].

In Low- or Middle-Income Countries (LMIC), when a person becomes bedridden or homebound due to chronic illness or injury, family members are likely to be tasked with caring for a dependent [2]. State involvement still needs to improve in such situations, but the local government (LG) driven primary palliative care programme in Kerala state, India, has been functioning for nearly 30 years as a well-acknowledged approach for community-based sustainable palliative care [3–5]. Governance in India comprises powers that are divided between a central government of the country, more regional state governments with separate legislatures, and local governments with locally elected representatives. Local governments oversee governance administration and developmental activities within specific jurisdictions like villages or towns, overseeing local infrastructure and services. Health is considered a subject of interest for the state governments. Kerala initiated decentralization reforms in several sectors including health care, where substantial funds and many functions were transferred to local governments [6]. The Kerala primary palliative care programme evolved with the support of local governments. Bedridden or homebound patients with serious health-related concerns requiring long-term symptom management are usually registered under this programme [5]. Pain and symptom management, psychological support for patient and family and provision of assistive aids and medicines are integral parts of the services rendered [3, 4]. However, even in this setting palliative care patients are highly dependent on others, primarily family caregivers, for their daily activities [4]. Family caregivers also help with medical and nursing care requirements [7]. Consequently, palliative care nurses often train family caregivers on simple and practical strategies of caregiving [4]. Thus, family caregivers play an integral role in translating programme services into better outcomes for the patient.

At times, for some such caregivers, this caregiving can become a burden, a multidimensional form of distress affecting their physical, psychological, social and financial well-being [2, 8, 9]. Perceived caregiver burden is associated with increased mortality, [10–12] poor health outcomes, including anxiety and depression [13, 14] and reduced quality-of-life among family caregivers [15]. Several studies have explored caregiver burden and associated factors [16, 17], but few studies have looked at these issues from the providers’ perceptive in LMIC [18]. Palliative care nurses have a limited understanding of caregiver burden and related issues. Patients remain the focus of care, while caregivers and their issues may go largely unnoticed [19].

Caregivers themselves may be sufferers of chronic diseases. This may be particularly true of Kerala, where the population aged 60 and above comprised 16.5% of the people in 2021 anisre expected to reach 20.9% by 2031 in Kerala [20]. Ageing caregivers may experience an increased impact of the consequences of caregiving along with physiological ageing, isolation and comorbidities [21]. With advancing age, multimorbidity is common among the ageing population [22]. Changing family structures due to migration and the increased number of women entering the workforce lead to many households having only ageing persons. Caring for a bedridden or homebound person by an ageing spouse is likely to be high in the Kerala population. Most such caregivers see ‘caregiving’ as their responsibility and feel obligated to provide care for their dependent. Spouse caregivers frequently report being more stressed and burdened compared to adult-child caregivers [9]. Ageing spousal carers may be at risk of increased cognitive impairment, loneliness, sadness, and anxiety compared to demographically matched ageing non-caregivers [23]. Also, our earlier analysis of depression among women caregivers had shown increasing odds of depression for higher age groups. These initial results underscore the significance of considering age as a potential factor that may contribute to varying experiences of burden among caregivers [13]. Age is usually treated as a confounder in studies on caregiving and adjusted at the time of analysis, and age-specific findings are not often reported [24]. Recently, however, research attention to the importance of ageing on caregiving outcomes is increasing [25]. There is a clear need to explore differences in experiences and needs of different age groups within the caregiver population so that targeted interventions and support strategies may be developed.

The World Health Organization in 2002 had recommended that services for chronic care should foster continuity of care and personal connection between the caregiver and the care recipient [26]. This will require functional relationships between the palliative care nurses and family caregivers, necessitating effective communication and rapport building by the nurse [27]. How the programme and its frontline representative, the palliative care nurse, perceive family caregivers, the caregiving role and caregiver issues are not adequately explored. A 2019 palliative care policy document from Kerala mentions caregiver support but this is still in a very early stage in the programme [28]. In this context, we studied the caregiver burden and quality of life of caregivers aged 60 years or above compared to younger caregivers of palliative care patients in Kerala. We also explored the perspectives of palliative care nurses on family caregiver issues in home care settings and whether these perspectives are reflected in the services offered by the nurses and the programme.

## Methods

### The palliative care programme

All panchayats in Kerala have a home care team that is led by a trained palliative care nurse. The nurse conducts periodic home visits along with the field staff of the local primary health centre, elected LG members and community volunteers. Each palliative care nurse schedules the home visits, directs patient health assessment and management and maintains several registers, one of which is the nominal register with patient name, contact information, diagnosis, and remarks on main service provision (e.g., catheter change, wound dressing etc.). We used the patient register of selected panchayats to identify patients and contact their caregivers for enrolment in the study.

### Sampling

The details of the sampling strategy for the cross-sectional survey have been published earlier [8].. The basis for sample size was adequacy for factor analysis– a sample size of 200 was deemed adequate for factor analysis with 25 items, achieving an item-to-participant ratio of at least 1:8 [34]. As male caregivers were very few, all male caregivers as reported by palliative care nurses were approached. Women caregivers were selected purposively from the list of patients in each panchayat palliative care registry to represent both cancer and non-cancer conditions.

Regarding sample size for the in-depth interviews, the primary objective of the in-depth interviews with caregivers was scrutiny of the representation of caregiver burden domains identified from the literature, and no new domains emerged after three interviews. For palliative care nurses, perceptions of caregiver burden were first identified and coded from literature and a draft thematic framework was prepared a priori. The first nurse interviewed belonged to the panchayats selected for the quantitative survey. During that interview, the interviewer (AK) felt that the nurse was fully aware of the caregiver issues encountered during the cross-sectional survey by the investigator and was giving responses conforming to the interviewer’s expectations. Therefore, four remaining nurses were purposively selected from panchayats in the same district that were not part of the quantitative study. Interviews were conducted to explore new categories and themes and data collection was stopped when no new categories emerged for two interviews.

### Design and data collection techniques

An integrative knowledge synthesis using mixed methods was carried out using analysis of a cross-sectional survey and qualitative exploration using in-depth interviews. This analysis used data from two study components done by the investigators, one on caregivers of palliative care patients and one on palliative care nurses. Table 1 summarizes the participant profile and data collection techniques for each study component.

**Table 1:** Participant profile and data collection of each study component

Participant profile	Data collection
***Study component - Cross-sectional survey and interviews with caregivers***	
221 caregivers of registered care recipients of primary palliative care projects from five panchayats in Kollam district, Kerala	Interview schedule with sociodemographic details, care recipient aspects, aspects of caregiving, caregiver burden (Achutha Menon Centre Caregiver Burden Inventory), quality of life (EuroQol EQ-5D-5L)
CG1*: 42 years old, caring for her mother, a 72-year-old stroke survivor, for the past 5 years	About role as a caregiver, things done in a usual day, overall experience, what they feel about their role as caregiver, effect on life, difficulties they face
CG2: 54 years old, caring for her 54-year-old husband, bedridden post-trauma 10 years ago	
CG3: 40 years old, caring for 80-year-old father-in-law, who had advanced lung cancer, bedridden for the past 6 months	
***Study component– In depth interviews with palliative care nurses***	
PN1*: 41 years old, 10 years of experience	Routine usual work routines, care practices during home care, interactions with caregivers, handling caregiver issues, caregiver support activities if any
PN2: 43 years old, 8 years of experience	
PN3: 48 years old, 9 years of experience	
PN4: 35 years old, 4 years of experience	
PN5: 41 years old, 10 years of experience	

* When reporting quotes abbreviation CG indicates Caregiver and PN indicates palliative care nurse

### Data collection from caregivers

The caregiver survey and interviews took place between January and February 2020. The investigators collected data for a study on developing and validating a Caregiver Burden Inventory in early 2020, published earlier [8]. The portion of that data used here comprised three in-depth interviews (IDI) with caregivers of palliative care patients and cross-sectional survey data of caregiver burden and related issues of 221 caregivers in five randomly selected panchayats in Kollam district, Kerala, India. This analysis focused on a comparison of findings of the cross-sectional survey on the caregivers aged above 60 years with younger or middle-aged caregivers aged between 18 and 59 years. All family caregivers of patients registered under the palliative care programme, aged 18 and above, who identified themselves as the primary caregivers and are providing care for not less than three months were included in the study. Those caregivers having a condition that limits their participation in the study and those caring for a critically ill care recipient during the study period are excluded from the study. An interview schedule was used to collect the sociodemographic information, care recipient and caregiver issues, and caregiver burden based on the Achutha Menon Centre Caregiver Burden Inventory, a nine-item inventory for assessing caregiver burden that had two domains– (i) physical, psychological, and spiritual aspects and (ii) financial aspects. Each item was scored on a 4-point Likert scale from zero to three. A caregiver could potentially score between zero (lowest possible burden level) and 27 (highest possible burden score). Quality of life also was assessed using the Malayalam version of the EuroQol EQ-5D 5-level version (EQ5D5L) [29]. We used the EQ-5D-5L Indian value set to convert responses to utility values [30]. The EQ-5D-5L is a widely accepted five-dimension HRQoL measure that covers mobility, self-care, usual activities, pain, anxiety/depression, and overall health state. It is easy to apply in younger and older populations and persons with less education [31]. It has good psychometric properties and the index values and dimensions have been found to strongly correlate with other measures of global health indicators, physical/functional health, pain, daily activities, and clinical/biological variables [32].

### Data collection from palliative care nurses

The researchers were part of a team working on decentralization and health in Kerala, in which one of the researched themes was the primary palliative care programme [33]. One of the themes selected for enquiry was caregiver issues. Five primary palliative care nurses (Table 1) with at least one year experience were purposively selected and interviewed to get an insightful account of their experiences with caregiver issues. Interviews were conducted telephonically due to COVID-19-related restrictions in 2020 and early 2021.

### Data analysis

To assess the validity of the EQ-5D-5L, we performed internal consistency checks and factor analysis of the five items of the EQ-5D-5L for the whole sample and the two age groups of interest separately (up to 59 years and 60 years and above). We extracted one factor from observed item values using principal axis factoring with direct oblimin rotation and correlated it with the utility scores obtained from the Indian value set of the EQ-5D-5L.

For the quantitative data, general characteristics and caregiver issues were summarised as frequencies and proportions or means and standard deviations, along with 95 per cent confidence intervals. Burden scores were converted to a categorical variable using tertiles, and labelled as low, moderate and high burden. Chi-square or Fisher exact tests were done to compare proportions. Analysis of variance (ANOVA) and posthoc Bonferroni tests were done to compare means. IBM SPSS version 25 was used for the quantitative analysis. Qualitative analysis was done manually.

All recordings of the IDIs were translated to English and initially coded by the same researcher (AK) who maintained an audit trail to map the interview transcripts and related codes to categories and themes. The approach to coding and categorising was inductive for the caregiver interviews and deductive for the palliative care nurse interviews. Information extracted from the literature review was used to generate a codebook for qualitative analysis to portray caregiver issues and perspectives of the nurse. The search was limited to articles in English, and title and abstract mention of caregiver issues along with provider perspective. Both investigators reviewed the shortlisted papers, and prepared codes, categories and themes through an iterative process. Existing codes were verified and additional codes, if any, were explored through triangulation with transcripts from caregiver issues mentioned by palliative care nurses in the main decentralization study. (See Additional file 1) After describing the findings based on this approach, we referred to Eva Kittay’s critique of Daniels and Nussbaum, based on the burden of caregiving and its effect on the caregiver’s opportunities while interpreting our findings from the study [35].

### Subjectivities of the researchers

AK conducted all interviews and both investigators were involved in the analysis and interpretations. Both investigators hold basic biomedical degrees and subsequently public health qualifications. The research experience of both researchers has been predominantly post-positivist. We believe that our experiences around epidemiological surveys would have shaped the data collection and interpretations in a predominantly biomedical perspective with some consideration of social determinants shaped by our experience level. However, our ongoing engagement with palliative care and caregivers’ issues also brings in some relational approaches and interpretations characteristic of literature on caring.

### Ethical aspects

All prospective study participants were assured of their autonomy, benefits and risks, privacy and confidentially and non-effect on care or benefits before obtaining informed consent. Informed consent, written or electronically documented, was obtained from all study participants. The Institutional Ethics Committee of the Sree Chitra Tirunal Institute for Medical Sciences and Technology, Trivandrum cleared all tools of the scale development phase. (Letter number SCTIMST/IEC/1444/NOVEMBER-2019 dated 14 November 2019). The proposal and tools of the palliative care nurse interviews, part of the decentralization project, were reviewed and cleared by the institutional ethics committee of Health Action by People Thiruvananthapuram. (IEC EC2/P1/SEP/2020/HAP dated 10 December 2020). While these were originally independent studies, clearance was obtained from the Institutional Ethics Committee of the Sree Chitra Tirunal Institute for Medical Sciences and Technology, Trivandrum (Letter number SCTIMST/IEC/2048/MAY-2023 dated 17 June 2023) for a synthesis exercise as part of formative research for a forthcoming study on caregiver burden assessment and intervention.

## Results

### Validity of the EQ-5D-5L in our study sample

We report the Cronbach’s alpha for internal consistency, the eigen value for the extracted factor, the factor loadings of the extracted factor onto each item of the EQ-5D-5L and Pearson’s correlation coefficient between the extracted factor and utility scores in Table 2. Internal consistency was moderate to good, eigenvalue was more than one and there was a high correlation between the factor derived from observed values and utility score values taken from the India value set. Factor loadings for pain/ discomfort and anxiety/ depression were relatively higher in the younger age group while for usual activities, factor loadings were higher in the older caregiver group.

**Table 2:** Findings of reliability and validity checks of the EQ-5D-5L in our sample

Variables		Younger or middle-aged caregivers (18 to 59 years old) (*n* = 159)	Older caregivers (60 years and above) (*n* = 62)	Overall scale (*n* = 221)
Cronbach’s alpha		0.681	0.679	0.708
Eigen value		1.734	1.596	1.801
Factor loadings	Mobility	0.624	0.698	0.696
	Self care	0.179	0.256	0.238
	Usual activities	0.233	0.520	0.382
	Pain/ Discomfort	0.836	0.652	0.801
	Anxiety/ Depression	0.748	0.581	0.687
Factor to Utility score correlation		-0.808*	-0.915*	-0.847*

*p < 0.001; Correlation is negative as high EQ-5D-5L item values indicate lower quality of life

### Findings from cross-sectional survey among caregivers

Palliative care recipients had various diagnoses ranging from stroke (23.9%), to cancer (12.8%) followed by other conditions. The mean age of the caregivers was 51.2 years (Standard Deviation (SD 12.7). The mean age of the older group was 66.2 years (SD 7.1) and of younger or middle-aged caregivers was 45.3 (SD 9.0). Caregiver ages ranged from 25 to 88 years. Demographic characteristic of the caregivers according to their age category is given the Table 3. Most caregivers were women, but in the older age group, the proportion of men was significantly higher. Older caregivers were significantly less educated and less likely to be married, but the social class was comparable.

**Table 3:** Sociodemographic characteristics of young or middle-aged and older caregivers from the cross-sectional survey

Variables	Categories	Younger or middle-aged caregivers (18 to 59 years old) (*n* = 159)	Older caregivers (60 years and above) (*n* = 62)	Chi-square p value
Sex	Male	11 (6.9%)	10 (16.1%)	0.04
	Female	148 (93.1%)	52 (83.9%)	
Highest educational attainment	No formal education	6 (3.8%)	13 (21.0%)	< 0.001
	Up to primary level	18 (11.3%)	18 (29.0%)	
	Up to higher secondary level	109 (68.6%)	27 (43.5%)	
	College degree and above	26 (16.4%)	4 (6.5%)	
Current marital status	Married	144 (90.6%)	50 (80.6%)	0.04
	Others	15 (9.4%)	12 (19.4%)	
Ration card type (Government allotted subsidy level)	Poorest of poor	19 (11.9%)	9 (14.5%)	0.74
	Below Poverty Line	89 (56.0%)	30 (48.4%)	
	Above Poverty Line– partial subsidy	26 (16.4%)	13 (21.0%)	
	Above Poverty Line– no subsidy	25 (15.7%)	10 (16.1%)	
Current employment status	Formally employed/retired	22 (13.8%)	13 (21.0%)	0.61
	Self-employed/others	54 (34.0%)	18 (29.0%)	
	Do odd Jobs	12 (7.5%)	5 (8.1%)	
	Not employed	71 (44.7%)	26 (41.9%)	

Table 4 depicts the distribution of variables related to caregiving. Nearly all caregivers in both groups were the sole caregiver for their care recipient. A significantly higher proportion of older caregivers were giving care to their spouses. Care requirements were significantly higher for the care recipients of younger caregivers, but most other variables were comparable. A higher proportion of older caregivers reported being satisfied with their caregiving activities.

**Table 4:** Caregiving aspects and caregiver burden of young or middle-aged and older caregivers

Variables	Categories	Younger or middle-aged caregivers (18 to 59 years old) (*n* = 159)	Older caregivers (60 years and above) (*n* = 62)	Chi square p value
Relationship to care recipient	Spouse	33 (20.8%)	38 (61.3%)	< 0.001
	Parent	12 (7.5%)	8 (12.9%)	
	Daughter/Daughter in law	104 (65.4%)	11 (17.7%)	
	Others	10 (6.3%)	5 (8.1%)	
Sole caregiver	Yes	156 (98.1%)	60 (96.8%)	0.86*
	No	3 (1.9%)	2 (3.2%)	
Current marital status	Married	144 (90.6%)	50 (80.6%)	0.04
Care recipient dependency level	Capable of selfcare	7 (4.4%)	6 (9.7%)	0.13
	Capable of limited selfcare	37 (23.3%)	19 (30.6%)	
	Fully dependent	115 (72.3%)	37 (59.7%)	
Medical diagnosis of care recipient	Cancer	18 (11.3%)	10 (16.1%)	-
	Cardiovascular disease	48 (30.2%)	19 (30.6%)	
	Old age/Dementia	32 (20.1%)	10 (16.1%)	
	Spinal injuries	18 (11.3%)	11 (17.7%)	
	Others	32 (20.1%)	12 (19.4%)	
	More than one medical condition	11 (6.9%)	-	
Duration in caregiving role	Less than or equal to 30 months	83 (52.2%)	28 (45.2%)	0.34
	More than 30 months	76 (47.8%)	34 (54.8%)	
Skilled care requirement	Not required	110 (69.2%)	51 (82.3%)	0.049
	Required	49 (30.8%)	11 (17.7%)	
Satisfaction with caregiving activities	No	136 (85.1%)	44 (71.0%)	0.01
	Yes	23 (14.5%)	18 (29.0%)	
Perceived Caregiver Burden	Low	70 (44.0%)	27 (43.5%)	0.95
	Moderate to high	89 (56.0%)	35 (56.5%)	
Subscale 1– Psychophysical and spiritual consequences of caregiving	Low	95 (59.7%)	31 (50.0%)	0.19
	Moderate to high	64 (40.3%)	31 (50.0%)	
Subscale 2– Lack of financial security	Low	57 (35.8%)	22 (35.5%)	0.95
	Moderate to high	102 (64.2%)	40 (64.5%)	

*Fisher exact test

Older caregivers reported poorer states for all variables related to self-reported morbidity and quality of life attributes measured using the EQ5D5L, except for self-care. (Table 5) Nearly three-fourths of older caregivers reported mobility issues; over half had pain or felt anxious or depressed.

**Table 5:** Self-reported morbidity and health-related quality-of-life variables of young or middle-aged and older caregivers

Variables	Categories	Younger or middle-aged caregivers (18 to 59 years old) (*n* = 159)	Older caregivers (60 years and above) (*n* = 62)	Chi-square p-value
Morbidity	No morbidity	92 (57.9%)	18 (29.0%)	< 0.001
	Any one medical condition	38 (23.9%)	18 (29.0%)	
	More than one medical condition	29 (18.2%)	26 (41.9%)	
EQ-5D-5L Mobility	No Problem	109 (68.6%)	17 (27.4%)	< 0.001
	Mild to Severe problem	50 (31.4%)	45 (72.6%)	
EQ-5D-5L Self-care	No Problem	155 (97.5%)	57 (91.9%)	0.14*
	Mild to Severe problem	4 (2.5%)	5 (8.1%)	
EQ-5D-5L Usual activities	No Problem	154 (96.9%)	53 (85.5%)	0.007*
	Mild to Severe problem	5 (3.1%)	9 (14.5%)	
EQ-5D-5L Pain	No Problem	105 (66.0%)	22 (35.5%)	< 0.001
	Mild to Severe problem	54 (34.0%)	40 (64.5%)	
EQ-5D-5L Anxiety/ Depression	No Problem	99 (62.3%)	27 (43.5%)	0.01
	Mild to Severe problem	60 (37.7%)	35 (56.5%)	

*Fisher exact test

**Fig. 1:**
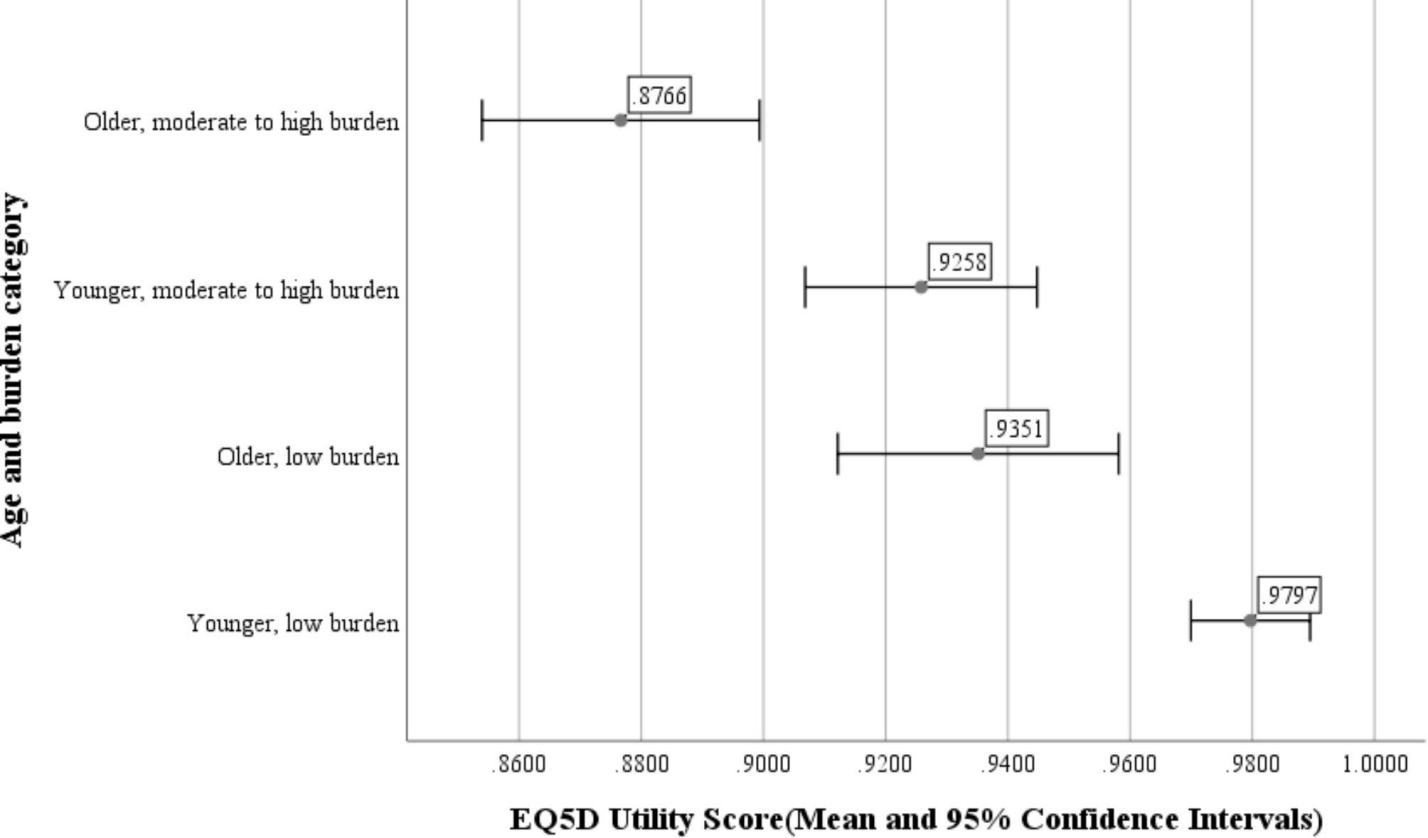
Means and 95% confidence intervals of EQ5D5L utility scores for caregivers grouped based on age category and burden level

The mean EQ-5D-5L utility score for the caregivers was 0.936 (SD 0.078, 95% CI 0.926–0.947). On comparing the caregiver’s age and burden experienced with the utility score, we found that the burden level impacted the perceived quality of life, irrespective of the caregiver’s age. As shown in Fig. 1, younger caregivers generally had a better quality of life than older caregivers, and those with low caregiver burden had better utility scores than those with moderate to high levels of caregiver burden. Younger caregivers who perceived a high burden level had lower mean utility scores (0.926, SD 0.090, 0.907–0.945) than younger caregivers who perceived a low burden (0.980, SD 0.041, 0.970–0.990). Likewise, older caregivers who perceived a higher burden level had a lower mean utility score (0.877, SD 0.066, 0.854–0.899) than their counterparts with a low burden (0.935, SD 0.058, 0.912–0.958). Except for the difference in means between older caregivers with low burden and younger caregivers with moderate to high burden, all mean differences were statistically significant. (*p* < 0.001)

Table 6 maps the support these dyads received regarding palliative care nurse visits, assistive devices, food kits or support from non-governmental charitable organisations. The frequency of nurse visits (monthly or above) was determined almost exclusively by patient need and was not associated with caregiver burden level. Among other forms of support, receiving food kits from the LG was found to be significantly higher when high levels of caregiving burden were present.

**Table 6:** Level of care recipient needs, caregiver burden, and nurse home visits from the cross-sectional survey

Patient needs skilled care	Caregiver burden	Number of dyads	Palliative care nurse visits at least once a month	Other forms of support		
				Assistive devices for care	Food kit	Support from NGO
Yes	Moderate to High	32	31 (96.9)	21 (65.6)	10 (31.3)	2 (6.3)
	Low	28	24 (85.7)	13 (46.4)	4 (14.3)	3 (10.7)
No	Moderate to High	92	52 (56.5)	25 (27.2)	26* (28.3)	7 (7.6)
	Low	69	36 (52.2)	18 (26.1)	6* (8.7)	2 (2.9)

NGO– Non-Governmental Organization

*p = 0.002

### Themes from in depth interviews with caregivers

#### “I do everything for her/him”

All caregivers mentioned doing “everything” for the care recipient, including all activities of daily living, medications, and procedures like skin care.

CG1: “I do everything for her…I bathe her… take her to the toilet…help her to change her dress…. Give her food. Everything…”.

CG2: “I’ve cared for my husband for the last 10 years. he is entirely dependent on me… everything…I clean him…bathe him… give him food…everything”.

#### Caregiving became physically and psychologically demanding

Doing “everything” involved physically demanding activities that reportedly led to chronic body pain for the caregiver.

CG2: “…a constant pain on my legs… I always lift him alone, there will not be anybody home…”.

Other issues mentioned included sleep deprivation, financial and job-related issues, and limitations to social participation due to caregiving. Care recipients could also have a temperament that made caregiving challenging.

CG2: “He is always very angry. He always shouts at me and my son… I’m always worried… I do not know what to do…”.

#### Care team is only patient-focused, caregiver issues are not addressed

The palliative care team when they visit would do patient-centred procedures, dispense medicines, and provide advice for improving patient care.

CG3: “…people from health (services) come once a month and change the urine tube…. They give medicines also…”.

Some advice provided could not be implemented, often due to affordability issues.

CG2: “…they give instructions about how to do physiotherapy…but it is no use…once in a month we used to call a physiotherapist…but it is expensive…”.

### Themes from palliative care nurse interviews

We shortlisted 17 articles for further analysis. Four were from Kerala and the rest were from outside India. Caregiver issues highlighted included burden, burnout, and health and wellbeing-related issues. Four themes on the care provider perspective were initially decided upon, namely: (i) exposition of caregiver burden by providers (ii) nature of family caregiver-health provider relationships (iii) factors that enable or hinder caregiver support from providers (iv) specific interventions that foster caregiver endurance.

Each provider interview took about 40 min, ranging from 35 to 50 min. Open codes from documents were binned into existing categories in the schema or new categories were added, if felt necessary. (See Additional file 1) No codes fell into the theme “specific interventions that foster caregiver endurance”. Brief descriptions of the findings were as follows:

#### Accurate exposition of the caregiver burden by palliative care nurses

All nurses highlighted the “burden” experienced by the family caregivers, mainly expressed as socioeconomic deprivation and challenges.

“Issues like no secure house, no food due to lack of income… patients who cannot buy expensive medication and continue their treatment… bystanders struggling for their children’s education…” (PN2– when reporting quotes abbreviation PN indicates participant attribute - palliative care nurse).

“They talk about the difficulties of not being able to go to work leaving their Amma (mother)” (PN1).

Added to this were disruptions and conflicts that the caregivers must handle along with the caregiving role.

Caregivers cannot sleep, they cannot look after their home and other household works, they cannot do their own activities like taking care of children (PN1).

Nurses often found themselves encountering conflicts, either between the caregiver and the patient or among family members taking the main caregiver responsibility. Sometimes patient behaviours were distressing for caregivers.

Sometimes patients will be so “violent” because of their condition; sometimes the patient’s condition is so bad… This also reflects on the caregivers. This affects them and they may also become frustrated. (PN1)

The caregiver role often limited the caregivers to their homes and restricted their social life. Societal perceptions of caring often deepened this social restriction. Nurses clearly described difficulties associated with long-term caregiving including physical pain, psychological distress, individual life disruptions, economic, and social challenges. Some caregivers had become sick from the long haul of physical exhaustion.

### I know caregivers like these…so desperate and hopeless… (PN5)

Nurses also felt that caregivers often neglect their well-being and prioritise their patient’s care.

#### Disparate relationships between caregivers and health providers and the system

Nurse representations of caregiver-provider relationships were complex, ranging from excellent cordiality to open conflicts. Nurses were at times “being like a family member” and at other times involved in verbal altercations and in extreme situations, involvement of law enforcement when neglect of the care recipient was perceived. A consistent part of the relationship, however, was the instrumental contribution expected from the caregiver in caring for the care recipient. Family caregivers were taken for granted as resource persons for caring for the patient and interactions mostly involved general instructions on caregiving or specific training for skin care, wound care, or catheter care. Some task-shifting often happened from the nurses to capable caregivers.

“We made them do these in front of us… The caregiver has taken care of the patient so well.” (PN1, mentioning an example of caregiver education for wound dressing).

Referral for palliative care itself might be perceived by family members as further care was largely up to themselves. It would often take multiple visits to discern all such concerns.

“…they also share their concerns… as palliative (is understood as) end-of-life care…so these makes them worried…” (PN5).

### The first time they won’t say everything… after numerous visits, they tell us everything (PN3)

When disagreements were encountered, nurses tried to resolve them by working for a healthy relationship between the caregiver and the care recipient. A somewhat stereotypical portrayal of caregiving emerged in the discourse, where caregiving was a moral imperative of the family, often women. The “best” caregivers were those who fulfilled this expected role well.

“I strongly believe that we should take care of our own parents” (PN2).

“There are no issues or problems for caregivers who are not working” (PN2)

“She is a widow…has two kids…the patient is her late husband’s mother…she (caregiver) is working… she does everything for her patient; only after that she leaves for work… When we visit the patient…it’s so clean and we never feel it’s a room of a bedridden patient…there are caregivers like this” (PN3).

Some caregivers were hesitant to build relationships with palliative care nurses. Nurses too might choose against investing time and visits for getting better acquainted with the caregiver. Caregivers who were demanding and making decisions independent of the nurse were considered problematic.

“They (caregivers) “torture” us by making calls to the panchayat member (the elected LG representatives who helm the programme)…” (PN4).

Families perceived as neglecting the care recipient were labelled as outright problematic. At times, nurses tend to establish an authoritarian role in such instances.

“I say to them if you did not take care of your parents, your seven generations will suffer…” (From additional codes as indicated in the additional file, said by a nurse based on the spiritual belief on results of bad deeds being passed on to future generations) (See Additional file 1).

“I say, “If you didn’t take care of them, I will inform to (the elected LG representatives) and doctor…If… your mother is lying in (urine and faeces), then you will be taken by police” (From additional codes) (See Additional file 1).

But palliative care nurses were often the first in the health system to recognize patient negligence and abuse by the family.

Caregivers who followed their instructions well and include nurses in treatment-related decisions were considered dependable. Yet, once good communication and rapport were established, caregivers often began to consider the nurse “like family” and this was highly valued by nurses, who mentioned several “friendships” that continued long after the death of the patient.

“(When her) daughter (finished school) she (caregiver) asked me which (field of education) is good for her daughter… now, following my advice, the daughter is doing nursing in the district hospital.” (PN3).

#### Systemic factors often hinder caregiver support

By systemic factors, we mean programmatic focus on the patient, lack of training, lack of time and limited attention to support schemes involving caregiver issues and burden. As such, there were no caregiver-specific initiatives or systematic documentation of caregiver issues. Caregiver support when existed was reactive rather than proactive. Caregivers were mostly given instructional support and/ or instrumental assistance for aiding patient care like medicines, cotton pads, gauze, catheters, Ryle’s tubes, or mobility aids. Communication and consoling were perceived as the main form of intervention by palliative care nurses.

“Their (caregivers) blood pressure will increase because of this lack of sleep. So, during our home visit we will check their BP also…” (PN1).

However, nurses informed eligible caregivers and families about beneficial schemes (‘*Ashwasakiranam*’, a state government-initiated financial assistance scheme for primary caregivers of palliative patients with cancer) or helpful charity organizations, if any.

Lack of time was the main impediment in addressing caregiver issues. Additionally, inadequate training and resources for giving caregiver support were also mentioned. Nurses suggested some systemic failures in recognizing the medical and social issues of caregivers.

“Some of the caregivers, have issues like CKD (chronic kidney disease), cancers or heart problems, but we cannot register them with the palliative care programme.” (PN5).

The main LG support specifically mentioning caregivers was the annual *Kudumbasangamom* (family gathering) with some recreational programmes, that too in the pre-pandemic days. Some LGs had schemes for self-employment generation for patients or caregivers, to make some products that could be sold for money. LGs support for hosting such schemes was patchy.

“But there was no adequate support from our panchayat for selling their product or purchasing the raw materials…no support for promoting these initiatives.” (PN1).

## Discussion

In this mixed methods study, we attempted to compare caregiver issues between older and younger caregivers in the palliative care program in Kerala. We also tried to document provider-side perspectives on family caregiver issues as articulated by palliative care nurses. The family caregiver issues we identified included physical, psychological, social, and financial issues, much like those reported by Ferrell and Wittenberg in their review of family caregiver trials in cancer patients [36]. As expected, older caregivers were more susceptible to health-related problems at this age. Irrespective of age, those who experienced a higher burden level had poorer quality of life. When combined, with higher burden experience, older caregivers had the poorest quality of life. This might be brought on by the physical demands of providing care as well as the ageing process’s effects on health.

The absence of any specific service or programme that enables caregiver endurance or any mention of systematic documentation of caregiver issues is a programmatic shortcoming. Nurses gave more attention to patients with skilled care needs and the level of caregiver burden was probably not a factor in determining their visits. Nurses’ tendency for “non-inviting interactions” with family members of patients, by prioritising medical and technical tasks, has been reported earlier from institutional settings [37]. But nurses recognised most caregiver issues and mentioned insufficient time to address them. Healthcare providers in similar programmes may not even have time for meeting their personal needs due to work demands [19]. Nurse perceptions about caregiving-related challenges mentioned social determinants of health but also mirrored prevalent socio-cultural and patriarchal norms. Family caregiver-centric studies are rare from LMIC, but available studies reflected socioeconomic deprivation and intense gender-role-driven concentration of caregiving in women [38]. Nurses however actively tried to improve the family caregivers’ skills in caregiving. This is important to prevent and delay burnout [39]. Additionally, they provide psychological support, often bonding well with caregivers long after they are bereaved [40]. Receiving interventions like food kits was significantly higher when the perceived caregiver burden was high. Caregiver burden is multi-dimensional and includes financial difficulties [8]. LGs generally focus more on the poorest and this finding is expected. The interventions remain basic, but it is promising that LGs can prioritise families with high caregiver burdens for interventions.

Poor households might disproportionately access the LG-run palliative care service, as the services are free of cost. Such households may already have high burden due to pre-existing structural and social disadvantages. Yet, even if caregiving was not causal for the problems expressed, the perceived burden would still be detrimental to quality of life. The directive principles of state policy of the constitution of India clearly list the fundamental rights of citizens and the responsibility of the state to protect citizens unable to access the minimal provisions for social and economic well-being. These principles also mention the autonomy of LGs [41]. It is thus a moral requirement of the LG-run palliative care programme to focus on the needs of families in addition to the patients.

Our findings draw attention to an important element of long-term care that is somewhat neglected– caregiver impact. Caregiving is a moral responsibility between individuals and at the collective level, as all individuals need care and are dependent at some point in their lives. But caregiving is a mix of reward and burden. Caregivers remain seen as a means to an end when in reality the caregiver is also an end in herself or himself. Allocation of caregiving responsibility is heavily gendered, rendering it as a form of inequity. Potential disadvantages of women may get compounded when she gets restricted to the caregiver role– lesser education, or work opportunities, and often treated as if she is unemployed or not doing economically productive work– leading to depression and a low sense of worth. Another aspect of caregiving that has implications for equity is the way society often works, based on normative or normal people. This may become unfair to suffering people as well as their caregivers, and the burden may be considered inevitable. The family caregiver is not a biological extension of the care recipient’s situation, to be moulded to sustain the biological functions of the care recipient. Neither is caregiving by a family member a law of nature that cannot be changed. This is a situation shaped by relationships between people and societies and the values and practices thereof. Moral requirements of caregiving should also consider what is lost to a caregiver and provide respect for the caregiver. Solutions may be explored by forming partnerships between the caregiver and others and by tapping into existing community resources. This has to happen without diminishing the relationship between the caregiver and the care recipient [26].

Norman Daniels proposes a lifespan approach of justice that may be useful to consider in this setting [42]. As individuals get older, their needs changes. When the society itself in an ageing society, that too brings in a new set of needs. In such a situation, reasoning has to be applied on how competing needs are to be met. Competing needs would be between different age groups or between care recipients and those giving care. Some needs would inevitable not be met when social obligations are to be met, but there should be fairness in the terms involved, and adequate social support to prevent issues like burnouts. Identifying beneficial interventions will remain an ethical challenge due to three aspects: (i) the vulnerability of the care recipient should not be exploited (Daniels); (ii) the voice of the caregiver has to be used for meeting the needs of the care recipient, as the capabilities of the latter have diminished (Kittay); (iii) the caregiver too has interests that would often be diminished (Kittay). The caregiver burden is disproportionately a woman’s issue because most of the caregiving work is rendered by women, many of whom are older persons. Discussions of fairness and equity often focus on fair distribution of goods like education and health. As Kittay points out in response to Norman Daniels and Nussbaum, conventional approaches to justice focusing on fair sharing of goods and aiming for equality of opportunity or capability do not talk about fair sharing of burden. In ageing societies, considerations of the distribution of burden may be as important as the distribution of goods.

The CARE framework refers to caregivers as “hidden patients” and recommends a framework comprising Caregiver well-being, Advanced care planning, Respite, and Education for planning to address caregiver issues [43] The first attribute in addressing family caregiver-related issues is an assessment of need. Symptom severity of care recipients, marginalized families and caregivers with significant psychosocial issues have been suggested as potential indicators of high caregiver issues [44, 45]. The deployment of tools like carer support needs assessment tool might help identify support needs and decrease caregiver strain [46]. Newer modalities like an app-based assessment are being tested in Sweden for family caregivers of patients with dementia [47]. Examples of successful caregiver interventions from LMIC countries are generally few. The trials covered in the review by Ferrell and Wittenberg were mostly from high-income countries [36]. In New Zealand three themes of advice for caregivers were considered most useful by providers– caring for oneself physically, emotionally, and spiritually; learning practical skills; and knowing what to expect and plan for as the family member’s health declines [48]. Researchers from the Netherlands recommended appreciation, information, practical support, and opportunities for time off (like respite care) as useful to lessen caregiver problems [49]. An intervention based on group sessions for caregivers in South Korea also showed promising physical and psychological outcomes [50].

Most of these examples are based on individual-level interventions. Krieger et al. reported the need for comprehensive caregiver support at two levels– the individual caregiver level, and the system level [51]. The United States of America (USA) has had several legislative and programmatic structures aimed at minimizing caregiver distress [52]. Caregivers of veterans in the USA have specific support like training, financial support, and assistance of a caregiver support coordinator, although Zebrak mentions about the lack of coordination between such policies [53, 54]. The National Health Service in the United Kingdom has some specific measures to support caregivers [55]. The National Institute for Health and Care Excellence, UK has included an assessment of caregivers’ quality-of-life in economic evaluation in its health technology evaluation manual published in January 2022 [56, 57].

The primary palliative care programme in Kerala is run by the LGs with support from the health department. Each LG unit sets aside resources from its annual fund allocation to support the wages of the palliative care nurse, travel costs, and costs of equipment, materials, and drugs for home-based care. Additional community-based resources are also mobilised by some LGs. In Kerala, the decentralized health system and the agency available with LGs for extending welfare measures to the needy using locally identified resources offers promise for good interventions [6]. Caregiver training and certification could be done, and a list of authorised paid caregiver schemes could be piloted, with efforts to include men in the initiative [58]. Facilities for respite care [59] may offer some personal space and time for caregivers, or additional appropriate practical help [60] could be offered. Building the competency of caregivers could extend to self-care in addition to patient care [39]. The formation of caregiver peer groups could be another intervention that facilitates information sharing, coping and increased social interactions [39, 61]. A specialist support service like a caregiver support coordinator or group could be initiated by the district-level health structures of the National Health Mission or the LG or by NGOs. Despite limited evidence of the success of such interventions on a large scale, it is useful to remember the economic value of family caregivers to the health system and community [45].

### Limitations

The quantitative data being cross-sectional, the temporality of the associations we saw cannot be ascertained. Poor health may cause poor quality of life and that may precipitate caregiver burden rather than burden resulting in poor quality of life. However, the implication for the health system remains somewhat the same– poor health, poor quality of life and high caregiver burden need attention whatever the order of their occurrence. Another limitation of the study is the lack of direct interaction with palliative nurses due to COVID-19-related restrictions. The interviews were possibly influenced by the previous experience of the researchers on caregiver issues. Physical visits to the settings and interactions with a wider group of stakeholders from the health department, the LG and other community representatives would have provided richer descriptions of caregiver issues and more quintessential details of caregiver-provider/ system interactions. At the analysis stage, we did not do a multivariable analysis to account for potential confounding or effect modification as data were not primarily collected to explore these aspects. The largely deductive qualitative analysis based on a priori themes is another limitation. As our focus was on validating our literature-generated construct of caregiver burden, we did not explore the experiences of elderly caregivers at that stage of the study and this is a drawback of this synthesis. Yet, we feel that our findings offer some insights that can be used to inform future research in this area.

## Conclusion

Caregivers aged 60 years or above made up three out of ten caregivers, with over half caring for their spouse, in this study setting. This is one of the first studies using Indian values of EQ5D5L utility scores for studying the quality of life of caregivers. Older caregivers reported a poor health-related quality-of-life and were experiencing a dual burden of caregiving and poor health, also having chronic health issues needing to take care of others while having to take care of others. The complex dynamics of caregiving by elderly caregivers have not been explored much, suggesting opportunities for future studies to explore these issues and develop targeted interventions for their specific needs. Potential interventions could be Respite care and support services for older caregivers that could offer temporary relief and help caregivers take breaks from caregiving responsibilities. Peer support groups could be another approach that can help caregivers to cope better with the burden. Also, comprehensive geriatric health and wellness programmes encompassing preventive, promotive, curative, rehabilitative and palliative care that jointly cater to patients and caregivers together are needed in settings with high ageing and chronic health conditions.

### Electronic Supplementary Material

Below is the link to the electronic supplementary material.

Additional File 1. Coding Schema for Coding Palliative Care Nurse Interviews.

## Data Availability

The corresponding author will provide the transcripts, data set and analysis of this current work on reasonable request.

## Abbreviations

ANOVA: Analysis of variance
CG: Caregivers
EQ5D5L: Five level EQ-5D version of EuroQol
IDI: In-depth interviews
LMIC: Low- or Middle-Income Countries
LG: Local Government
PN: Palliative Nurse
SD: Standard Deviation
UK: United Kingdom
USA: United States of America
