# Supplementary material for: Understanding caregiver burden and quality of life in Kerala’s primary palliative care program: a mixed methods study from caregivers and providers’ perspectives
Source: Int J Equity Health. 2024 May 7;23:92. doi: 10.1186/s12939-024-02155-x (PMC11077822; doi:10.1186/s12939-024-02155-x)
Supplement: Supplementary file 1 — Additional File 1. Coding Schema for Coding Palliative Care Nurse Interviews. [file 12939_2024_2155_MOESM1_ESM.docx]

**Understanding Caregiver Burden and Quality of Life in Kerala's Primary Palliative Care Program: A Mixed Methods Study from Caregivers and Providers' Perspectives**

**Additional File 1 - Coding Schema for Coding Palliative Care Nurse Interviews**

Codes without citations were developed from caregiver-related issues emerging from transcripts of interviews the investigators conducted as part of a study on decentralization and health in Kerala.^1^ One area of focus of the study was the primary palliative care programme in Kerala where 28 in-depth interviews were conducted, 14 of which was among palliative care nurses (PN).

1. **Exposition of caregiver burden by providers**
   1. Caregiver burden
      1. Financial burden^2^
      2. physically demanding^2,3^
      3. emotional/ psychological^2,3^
      4. sleep deprivation^2^
      5. no leisure times
      6. socially restrictive^3^
      7. Income loss
      8. Family conflicts,
      9. Deterioration of mental health^4^
      10. Self-neglect^5^
      11. Spiritual and religious issues^6,7^
      12. Role conflict (family)
      13. Caregiver – unaware of patient condition
      14. Lack of family support
      15. Adequate Family support
      16. Abusive care recipient
      17. Women are expected to be caregivers
      18. Exclusion^8^
      19. Forced to be caregiver by family
      20. Sole caregiver
      21. Caregiver- morbidity and related issues
      22. Caregiver strain and stressed out
      23. Anxious and worried about themselves and their care recipient
      24. Become sick because of caregiving role
2. **Nature of family caregiver-health provider relationships**
   1. Caregiver as a resource person^8^
      1. Teach to provide care and support – e.g., adherence^3,9,10^
      2. Task shifting – medical tasks like wound dressing^3,10,11^
      3. Specific training^3,12^
      4. Dependable caregiver - Stick to instructions and advice
   2. Attitude to caregiver
      1. Stereotyped image of caring^12^ (a person caring for a loved one, who needs to be taught, or is a person to aid speedy recovery of patient)
      2. Expected role of caregiver
      3. Care giving - duty and responsibility of caregivers
   3. Partial focus on caregiver
      1. Primary focus on patient
      2. Low awareness of ill effects of caregiving
      3. Allay caregiver apprehensions^10^
      4. Grief and bereavement support - psychological support nearer to end of life or after patient expires^13^
      5. Caregivers actively discuss about patient (treatment related) not about themselves^14^
      6. Extending care more than ‘general care’
      7. Better care for patient by caregivers
   4. Complex relationships
      1. Conscious ignoring from both sides^14^
      2. Conflicts^14^
      3. Friendships that continue after patient’s death^14^
      4. Bonding is time consuming^14^
      5. Consider like a Family member
      6. Caregivers as burden (independent decision and demands)^8^
   5. Roles played by PN
      1. Building healthy relationship between caregiver and care recipient
      2. Role in family decision making
      3. Nurses act a link between charity key persons and caregivers for family needs of the caregivers
      4. Patient neglect
      5. Psychological support/Listeners
   6. PN – an added pressure
      1. Nurses is forcing to take up the caregiving role
      2. Nurses threatening the caregivers
3. **Factors that enable or hinder caregiver support from providers**
   1. Mostly institutional/ instrumental; less of psychological/emotional^3,12^
      1. ‘Personal’ time of provider^12,14,15^
      2. No caregiver specific care
      3. Institutional
      4. Instrumental
   2. Support is reactive, not proactive^15^
      1. Documentation of caregiver aspects is lacking^13^
      2. Do recognise the caregiver issues^8,12,15^
   3. Lack of specialist carer support services (e.g., caregivers of mentally ill patients)^8^
      1. Inadequate training/recourses for nurses^8,14,15^
      2. Provider centric model without involvement of caregivers and users^11^
      3. Providing the service as per capability and resource for caregivers
      4. First referral in palliative care
      5. Health system barrier for medical support for caregivers
   4. Panchayat (Local government) support
      1. No specific projects for caregivers from panchayat
      2. Employment generation for patients and caregivers
      3. Lack of interest from panchayath
      4. Supportive panchayat
      5. Assistive aids for patients
      6. Power hierarchy and barriers from panchayat
   5. Support for family
      1. Support for family – Educational
      2. Support for family – Financial
      3. Support for family – Food kits
   6. Specific programmes for CG and patient
      1. Recreation for patients/caregivers
      2. Employment generation for patients and caregivers
4. **Specific interventions that foster caregiver endurance**
   1. Build peer groups to overcome this^16^
   2. Competency building^16^
      1. Knowledge, preparation^16^
      2. Self-efficacy
         1. Personal space and time^16^
         2. Seek treatment and social security measures^16^
      3. Potential utilization as formalized certified caregivers.^17^
   3. Practical help^15^
   4. Respite care^15^

**Quotes from palliative care nurse interview transcripts of the main study^1^ that were listed as part of triangulation of five in depth interviews of this synthesis**

“Once there was one person who never takes care of her mother and we went there with Police. Such an experience is there. She was the only daughter for that mother” **(PN6;5,6)**

“Difficulties for the caregiver… like sleeplessness…” **(PN6,46)**

“… then we will put the tube … and show to the family members, if they need to replace the tube, then monthly we will replace it.” **(PN7;53,54)**

“We conduct *Kudumba Sangamam* (family gathering) every year. For those patients who can come, then they will come for these *Kudumba Sangamam*. If they cannot come, then the bystanders will come.” **(PN7;59-63)**

“Rehabilitation schemes were available like making disinfectant solutions, we gave some materials to teach them how to make etc” **(PN7;65,66)**

“Sometimes because of the medical condition of the patient, other family members may be suffering like if the son is bedridden and his father and mother are there.” **(PN7;68,69)**

“The main activities we done are first tours, second teaching how to make candles, soap etc, third one, which is to be attended mandatorily is called “rogee bandhu sangamom” - It is a meeting between the patient, bystanders, care takers and we Palliative staff.” **(PN8;87-89)**

“Cancer patients who have completed Chemotherapy and cannot go outside to prevent infection, and for those patients who are doing dialysis, they can take book from the library; read and return back to us. At that time, nobody was going outside and so everyone enjoyed reading books. We have a Whatsapp group with volunteers also and we share the names of book, content and such details in the group.” **(PN8,102-106)**

“The patient cannot do this by himself. So, we will start our care from hair of the head till the nails of the leg. Start from cleaning and training will be given to the bystanders. When they see these things for the first time, it will be very difficult for the bystanders as they have a doubt that whether these things can be done by them.”  **(PN9;115-119)**

“We cannot go there daily and do the things for the patients. So, we will do and teach them how to do.”  **(PN9;120,121)**

“Mainly they are afraid of the patient’s condition.” **(PN9;193,194)**

“So, with our behaviour, we changed the behaviour of many family members of the patient. None of the Palliative Trained nurses can leave a patient in such a condition after the training they received from palliative care.” **(PN9;198-201)**

“In some houses, they (caregivers) will be waiting for us to come. When we miss for one month, they will enquire about us.” **(PN10;206-207)**

“Then, we showed them and we demonstrated for them and we provided them all the things for dressing the wound. We also told them that if you cannot do, inform us and we will come and assist you in dressing the wound” **(PN11; 224-226)**

“His mother considers me as a daughter” **(PN11;237)**

“Some caregivers say that these people (nurses) are coming to shout on us.” **(PN11; 257,258)**

“If we say positive things, then they may consider cleaning the patient better. Those may not be the required things to be done, like that they may start taking bath of the patient and such things. Initially, they may not be doing much. But once we say like this, even though if they are doing or not doing, we never told bad words. We told only good things. So, they will try to do more work” **(PN11;262-266)**

“They will clean not only before our arrival, but also at all times. In the morning, they do all those things which need to be done. If anybody is leaving, then another person will be kept in the house as a replacement” **(PN11;269-271)**

“Everybody calls us and ask doubts. Whenever any minor changes are observed, they ask us. There is slight change in colour, what to do? Like that. So, we clearly explain to them and they can prevent from becoming worse.” **(PN11;283-285)**

“We teach the lady members there on how to clean and the cleaning of private areas…” **(PN12;296,297)**

“…go to any bedridden patient, we show them how to do sponge (bath)… you have to clean like this. Thus, we teach the family members” **(PN12,293,294)**

“There are no such cases where the caregivers are not taking care of the patients. We inform the bystanders and they will do.” **(PN12;304,305)**

“Otherwise, I say to them if you did not take care of your parents, your seven generations will suffer, like that…So if we say like this for one or two times, then they take care of them very well. I used to frighten them…If you didn’t take care of them, I will inform to Panchayath member and doctor… If at that time, if your mother is lying in human waste, then you will be taken by Police …So, if we say to those people who did not take care of the patient, then they will take care of the patient. But there are very few cases…” **(PN12; 306,316)**

“Most of the old men and women, who are bedridden patients, will be in an angry mood” **(PN13,325,326)**

**References**:

1. Kochuvilayil A, Rajalakshmi S, Krishnan A, Vijayanand SM, Kutty VR, Iype T, Varma RP. Palliative care management committees: a model of collaborative governance for primary health care. Public Health Action. 2023 Mar 1;13(1):12-8.

2. Gater A, Rofail D, Tolley C, et al. “Sometimes It’s Difficult to Have a Normal Life”: Results from a Qualitative Study Exploring Caregiver Burden in Schizophrenia. Schizophr Res Treatment; 2014. Epub ahead of print 2014. DOI: 10.1155/2014/368215.

3. Philip RR, Venables E, Manima A, et al. “Small small interventions, big big roles”- a qualitative study of patient, care-giver and health-care worker experiences of a palliative care programme in Kerala, India. BMC Palliative Care 2019; 18: 16.

4. Schulz R, Sherwood PR. Physical and Mental Health Effects of Family Caregiving. Am J Nurs 2008; 108: 23–27.

5. Wingham J, Frost J, Britten N. Behind the smile: qualitative study of caregivers’ anguish and management responses while caring for someone living with heart failure. BMJ Open 2017; 7: e014126.

6. Vigna PM, de Castro I, Fumis RRL. Spirituality alleviates the burden on family members caring for patients receiving palliative care exclusively. BMC Palliative Care 2020; 19: 77.

7. Shin S, Huddleson M, Brown LM, et al. The role of religion in caregiver burden and depression for family caregivers of dementia patients. Innov Aging 2017; 1: 139.

8. Benzein E, Johansson B, Saveman B-I. Families in home care – a resource or a burden? District nurses’ beliefs. Journal of Clinical Nursing 2004; 13: 867–875.

9. Philip RR, Philip S, Tripathy JP, et al. Twenty years of home-based palliative care in Malappuram, Kerala, India: a descriptive study of patients and their care-givers. BMC Palliative Care 2018; 17: 26.

10. Sankaran BM, Chakraborty S, Patil VM, et al. Burden and Outcomes of Pressure Ulcers in Cancer Patients Receiving the Kerala Model of Home Based Palliative Care in India: Results From a Prospective Observational Study. Indian J Palliat Care 2015; 21: 152–157.

11. R Kottai S, Ranganathan S. Task-Shifting in Community Mental Health in Kerala: Tensions and Ruptures. Med Anthropol 2020; 39: 538–552.

12. Useros MVD, Espín AA, Parra EC, et al. Family Caregivers: Nurses_ Perception and Attitudes. Social Medicine 2012; 6: 151–161.

13. Gerrish K. Caring for the carers: the characteristics of district nursing support for family carers. Primary Health Care Research & Development 2008; 9: 14–24.

14. Salin S, Kaunonen M, Åstedt-Kurki P. Nurses’ Perceptions of Their Relationships with Informal Carers in Institutional Respite Care for Older People. Nurs Res Pract; 2013. Epub ahead of print 2013. DOI: 10.1155/2013/967084.

15. Simon C, Kumar S, Kendrick T. Who cares for the carers? The district nurse perspective. Family Practice 2002; 19: 29–35.

16. Solli H, Hvalvik S. Nurses striving to provide caregiver with excellent support and care at a distance: a qualitative study. BMC Health Serv Res; 19. Epub ahead of print 27 November 2019. DOI: 10.1186/s12913-019-4740-7.

17. Emanuel N, Simon MA, Burt M, et al. Economic impact of terminal illness and the willingness to change it. Journal of palliative medicine 2010; 13: 941–944.
